# New Jersey State Dental Society

**Published:** 1891-04

**Authors:** 


### NEW JERSEY STATE DENTAL SOCIETY.—TWENTIETH ANNUAL SESSION.

(Continued from page 182.)

*Thursday, July 17, 1890.—Morning Session.*

The secretary called the roll.

On motion of Dr. Sanger, it was "*Resolved*, That a committee of three be appointed to take into consideration the suggestions made in the paper of Dr. J. A. Osmun, entitled 'From Another Stand-Point.'"

The following resolution was offered and carried:

"*Resolved*, That the State Dental Society of Pennsylvania be invited to meet with this Society in joint session at our next annual meeting in July, 1891."

At the conclusion of the routine business of the morning session the President called on Professor Mayr, the next in the regular order of essayists. The professor stated he had not prepared a written paper, as he preferred to give his views extemporaneously and then answer questions.

### ACIDS AND ALKALIES—ACIDITY AND AVIDITY.

BY PROFESSOR CHARLES MAYR, SPRINGFIELD, MASS.

The fact that I am again allowed to address this Society is to me a very great encouragement. It is very easy for almost any one to address a meeting once, but to address the same association a second time is a very difficult and different matter.

I shall strive to do my best to present a subject which in this voluptuous atmosphere will not be too great a strain upon the mental effort to be put forth to understand it, and at the same time shall not be stupid. With this idea in my mind, I shall present to you an old, well-worn subject,—namely, "Acids and Alkalies."

Everybody knows everything about them, so that I have before me the pleasant and agreeable task of presenting to you only the well-known memories of scientific childhood and those things which you have all been familiar with for a long time.

Most of you may reasonably be supposed not to be special chemists, and, as you all know, chemistry, as taught to non-chemists, is very different from what the chemist himself sees and finds.

For instance, I am unable to give you the exact definition of an acid or an alkali, because, while the extremes are well pronounced, there are so many intermediates that the line of demarcation vanishes. The only definition I can give to you sounds ridiculous, —namely, a strong acid is one which is completely neutralized by a strong alkali, and a strong alkali is one which is completely neutralized by a strong acid. Certain superficial methods of recognition do not furnish in reality a good criterion, because they are not good in all cases. Circumstances alter cases so much. That which is an acid under one condition, exhibits characteristics of an alkali under another condition. For instance, there exist compounds of sulphuric acid with acid radicals, in which these acid radicals appear as alkalies, and, while not completely neutralizing the sulphuric acid, they at least diminish its acidity.

Take an alkali like oxide of zinc,—when combined with sulphuric acid to sulphate of zinc, the sulphuric acid is not completely neutralized; the salt reacts acid to litmus. This oxide of zinc combines also with alkalies, and there plays the part of an acid. The alkali is again not neutralized, but its alkalinity is slightly diminished.

There is a group called manganites, in which the peroxide of manganese appears as an acid, while there exist salts in which it appears as an alkali. I do not think the term "acid" is one which is susceptible of exact measurement. The distinction between acids and alkalies is somewhat based on a very uncertain sense,—that of taste. We have not been able to measure taste as we can measure light. Take picric acid, for instance: it has not the least acid, having only a bitter taste, and yet it is a very powerful acid. Among alkalies we have ammonia, which has a very powerful taste, while magnesia has very little such taste; yet the latter is a very much stronger alkali than ammonia; so that our common impressions are liable to lead us into the idea that there is very much greater difference than there is in reality. Our senses were evidently not made to be measuring instruments. Their purposes are those of every-day drudgery, to run the daily course of life.

For the sake of convenience we may group acids into five classes:

1. Very strong acids.
2. Diluted strong acids.
3. Strong organic acids.
4. Weak and doubtful acids.
5. Acids which do not show their acidity by simple tests.

To the first group belongs the well-known trio, sulphuric, hydrochloric, and nitric.

To the next group, phosphoric, the thionic acids, brom-hydric, and iod-hydric acids.

To the group of strong organic acids, oxalic, butyric, citric, picric, tartaric, lactic, and many nitro acids.

To the group of weak acids, carbonic acid, sulphydric acid.

To those which give not sufficiently the acid reaction, boracic acid, silicic acid.

Some acids which seem to be very strong at first investigation, prove very weak chemically. For instance, hydrofluoric acid is only one-fifth as strong as oxalic acid; sulphuric acid only one-half as strong as nitric; acetic acid only one two-hundredth as strong as muriatic, and only twice as strong as carbonic acid.

And just here I will remark that the fact of the existence of very weak acids chemically, while strong to the taste, is a subject which I do not think dentists have sufficiently considered; because the original investigation about them was published in a purely chemical work by Professor Thompson. He has investigated what he calls "avidity." We have all heard of affinities, but avidity is a subject which has not been familiar enough to us, although it is of great importance. Suppose I were to mix some sulphuric and acetic acid together. I put a little alkali in the mixture, not enough to neutralize them both. The question now is in what proportion will that insufficient amount of alkali be seized by the acids present. It will be seized in proportion to their "avidity." So the avidity gives you to a certain extent the measure of the activity of an acid under certain circumstances. This avidity of acids can only be obtained relatively. The acid which has the strongest avidity is muriatic acid, and almost equally as strong is nitric acid. If I mix a pint of muriatic acid and a pint of nitric acid and a quarter of a pint of alkali, the two acids would seize the alkali in equal proportions. But suppose I take sulphuric acid, which is not as avid as muriatic or nitric acid, having only fifty-nine one-hundredths of the avidity of muriatic or nitric acid. If I were to make a mixture of muriatic and sulphuric acid, and put an amount of alkali into it insufficient to neutralize it, two-thirds of this alkali would be seized by the muriatic acid, and only one-third by the sulphuric. So that in this fluid, besides the two acids in excess, there will be twice as much chloride (speaking of equivalents, not by absolute weights) as sulphates. Oxalic acid has two-thirds the avidity of muriatic acid, and on the other hand acetic acid (the acid of vinegar), which is very biting, indeed, has absolutely no avidity compared with these acids; that is to say, if I were to put one thousand pints of acetic acid with one pint of sulphuric acid, and put in just enough alkali so as not to neutralize both, every atom of sulphuric acid would have to be neutralized before the first atom of acetic acid could be neutralized by the alkali. So, if we were to mix one hundred pints of the strongest citric acid and one pint of muriatic acid with the proper amount of alkali, again, every drop of muriatic acid would have to be saturated by alkali before one drop of the citric acid would be allowed to combine.

This side of acid questions is just beginning to be considered properly, and in dentistry I think it is one of very great importance. Yesterday the question of erosion came up. The acid which causes this erosion (if it is caused by acid), or, I will say, the acid which *might* cause this erosion, certainly acts in proportion to its avidity. That is to say, suppose we have an acetic acid. This is a volatile acid, and is much weaker than a non-volatile acid. So the acetic acid has less avidity than lactic acid. Hence, if there was a mixture of lactic and acetic acid, the lactic acid would seize the lime before the acetic acid would act upon it. If you expose a piece of marble to the action of acetic acid, it will be dissolved in a crumbling manner, while if you expose it to the action of muriatic acid, it will remain smooth on the outside and become quite polished; and if you expose it to the effects of an acid the avidity of which is somewhere between these two, it will also be eaten smoothly. As a rule, in teeth a smooth and not a crumbling erosion is found, so that acetic acid is certainly not one of the acids causing that erosion.

The exact avidity of many organic acids has not been determined, because of the difficulty of separating the products.

This avidity explains also in a very satisfactory manner the reactions.

If we distil salt with sulphuric acid to obtain hydrochloric acid, the process is one of avidity more than affinity.

I do not care to go into the question of avidity any further, because it may not strike you, as dentists, as a matter of interest, although to us chemists it is a matter of very great interest. So much, therefore, for the matter of avidity.

Now we come to another aspect, that of acidity.

Acidity is a matter of our sense of taste, and of nothing else; that is to say, if you put ten thousand drops of water with one drop of sulphuric acid, you will perceive some acidity should you place it upon your tongue, while, if you mix together ten thousand drops of water and one drop of acetic acid, you cannot taste it at all, although, strange to say, you can smell it a little. If you mix ten thousand drops of water with one drop of oxalic acid, you can just about detect the presence of acid by taste. That is acidity. I do not think we have any other measurement for the acidity of an acid. The *affinity* of an acid (which is a term also frequently used) implies far more force than activity or avidity. By the affinity of an acid we understand its potentiality under given circumstances to combine with other chemicals,—that is to say, the possibility of a substance to combine. When speaking of affinity the circumstances of the experiment are of the greatest interest. To illustrate. Take silicic acid, which is not subject to the test of taste; place some silicic acid with some sulphate of soda in a crucible, heat it and wait until the soda melts, and the sulphuric acid comes off in great quantities, and finally every trace of sulphuric acid will be expelled by the silicic acid. The affinity of the acid has been greatly increased; the avidity, I presume, has also been increased; the acidity has not increased at all. So with boracic acid. I doubt if by its taste it can even be called acid, but if it is melted it is found to have very great avidity and affinity. So it is with a great many other acids; but I will not go into details, as I do not wish to be tiresome.

Now, as to the tests in the mouth for acids. Yesterday litmus paper was suggested, and various methods were spoken of in which to get a good, sensitive litmus paper. Litmus is excellent for most purposes, but it is too easily affected by carbonic acid, which will turn it quite red. It is also affected very much by the vapors of ammonia in the air, which very likely reduce its sensibility. For that reason I consider litmus paper somewhat treacherous in some tests. The best test is an alcoholic solution of carmine, which has to be applied of itself; you cannot make paper of it. Carmine turns yellow with an acid, and a purplish red with an alkali. There is a chemical substance (phenolphthaleine) which is perfectly colorless with an acid, and wine-purplish with an alkali. This can be used on paper. Ammonia affects it but little. There are a large number of similar test-papers which have been made out of aniline dyes, which are very useful. I have made this matter a special subject of investigation, so as to produce a test-paper which dentists can utilize for the various chemicals they use in their laboratories. For instance, a dentist wants to know whether his hydrogen-peroxide is still good or not. The best test is a paper made with a little iodide of potash, a little starch, and bicarbonate of soda, the mixture being smeared upon ordinary blotting-paper. So long as the hydrogen-peroxide is good it will produce blue spots on that paper, and when it produces only a purplish spot, or no spot at all, it is worthless. That is a simple test, and this paper will keep an indefinite length of time. For the mixture I take five grains of iodide of potash, five grains of starch, and half a pint of water, with a small pinch of bicarbonate of soda, and put the mixture upon any kind of paper that will soak it up, and so long as your hydrogen-peroxide is good it will produce a blue spot upon the paper. I have found this a very valuable test-paper. Then, for the various kinds of acids that you meet, an excellent paper is a decoction of logwood. With most of the chemicals this will turn different colors; with an acid, bright red; with an alkali, purple; ordinarily it is a kind of brownish red, and I consider it very useful, indeed. With a logwood test you can tell ten times stronger acids than with the litmus. A volatile acid on such a paper produces a reddish halo around a bright-red spot. A non-volatile acid produces only the spot with no halo, because no acid vapors spread around it. The test-papers are, I think, very valuable for practical purposes, and you can very easily make them, and with weak and strong acids and alkalies the indications are very good and valuable. Among other substances, aropæoline, for instance, is very useful; with an acid it is yellow, and with an alkali it is red, and the change is very prompt and very striking. You have probably all of you seen the experiment where a man pours water from a pitcher into a tumbler, and the moment the water touches the tumbler it is turned into red wine; then he takes the tumbler and pours the wine back into a pitcher; it is perfectly plain, you can see that it undoubtedly is red wine. But the moment afterwards he pours it out of the pitcher as water again. This is done by the use of penolphthaleine, which is perfectly colorless and as clear as water; but when mixed with alkali it becomes a fine purplish-red. So when you spread it upon paper in the presence of an alkali it will show bright-purple spots. On these papers you can also test chemicals that belong to the organic alkaloid group,—namely, morphia and such chemicals.

As a rule, I do not prepare a long paper, because it is impossible for me to ascertain beforehand what will meet with the popular demand, and I should be apt to bore the Society with general technicalities and complicated technical matters, because of not knowing exactly what you would like to have me tell you. I prefer to have the gentlemen present ask me any amount of questions, for that will probably bring out much better just what you would like to have me tell you, and will be of far more benefit than an hour's talk upon general matters. I should, therefore, like to be asked some questions.

*Dr. G. E. Adams.*—In what proportion would you mix the iodide of potassium and the starch?

*Professor Mayr.*—About three grains of iodide of potash, three grains of starch, and about one ounce of water. That will give a very fluid combination, and when spread on some kind of reception paper will make a first-rate test. Whenever you get a violet spot, the peroxide is of questionable value, and when blue it is very good.

*Dr. G. E. Adams.*—Must this paper be fresh every time it is used?

*Professor Mayr.*—No; you can keep that paper for a long time. It will turn slightly purplish, but not enough to interfere with the reaction. If that purplish tint should be objectionable, you would only have to add a little bicarbonate of soda. In certain atmospheres the paper will take a bluish tint; for instance, near the ocean, the ozone and the peroxide of hydrogen, which are found in the air, turn the paper blue, and after a while it will become quite a dark blue; still you have no difficulty to distinguish the result when making the test, as very much stronger spots are produced than the general darkness of the paper.

Now, in regard to alkalies. Perhaps that is a slight hobby of mine. I did not dare present it under its heading; but if the gentlemen are not too tired, I would like to speak of it here.

We have the organic alkalies, the so-called ptomaines, about which I once read a paper in Boston, and I was very sorry to see that because I gave it under its true title it was not properly appreciated. These organic alkalies are of much greater importance to us than lime, ammonia, and all those common alkalies. I had occasion, about five years ago, to translate the work of a German chemist, Dr. Brieger, on this very subject. He had succeeded in isolating the poison which is found in hydrophobia. The poison which causes the symptoms of hydrophobia is an alkali, and that alkali is manufactured by the microbe which produces the hydrophobia. I have since made a number of experiments, also to see if I could not get some of these alkaloids. I have succeeded in producing a few of them described by Dr. Brieger, but the work is extremely complicated and very difficult. As a rule, in order to make these experiments, a part of the body has to be exposed to the action of the microbes from a pure culture.

For instance, you may take the liver, the heart, etc.; these organs must be exposed to the culture of the microbes for from six to eight weeks, and gentlemen with lively imaginations can readily understand that this kind of work is one that should be conducted with open windows. These poisons, when produced, are most remarkable. The following have been well isolated: That of typhoid fever; that which produces in summer very frequent and very disagreeable complaints after eating ice-cream, stale milk, and stale cheese, etc.; that of poisonous mussels. As I have said, the poison of hydrophobia has been isolated. I recently had the pleasure of reading a very interesting paper by a physician who furnishes proof that life can be killed only by life. Take, for instance, the disease of diphtheria; this has been treated very many times by various applications, but the remedy for this disease seems to be erysipelas; the microbe of erysipelas, in a number of well-observed cases, entirely destroyed the microbe of diphtheria. In the experience of this physician, he found that by the application of this remedy he produced erysipelas to a slight extent, but it stopped developing all at once, and in a few days the patient under treatment rose quite well and quite rid of diphtheria and erysipelas. The two microbes had killed each other; he persisted in his theory and inoculated a number of persons suffering from diphtheria with the microbe of erysipelas. The affection apparently started and then disappeared, while the diphtheria also declined, and he has lost but one single patient out of a large number of cases treated in this way. This is a solution which I have always dreamed of in the field of microbic investigation. Life can only be killed by life; I don't think we can kill life by the application of inanimate drugs. My belief and my line of philosophical search certainly would not lead me in that direction.

As was suggested, in order to reduce decay we should kill the microbe of lactic acid which produces the decay; the portion of the tooth which has been destroyed during the process of its activity cannot be recuperated. I do not think we have even the slightest hint which will suggest to us any way in which lost tissues can be recuperated. The man who can tell us how decayed matter can be restored has found the fluid of eternal youth, the elixir of life; for the same principle which will recuperate teeth tissues will rejuvenate the whole person from head to toe. The secret, therefore, of recovering lost tissue is, in my opinion, not yet accessible. I even doubt if we are in a position to investigate that subject, and we should therefore direct our attention to a much easier problem of how to ward off an injury. There seems to be a well-settled principle that there are two contending forces everywhere in life, the one attacking from within and the other from without; but this subject is not, it seems to me, sufficiently realized. If we could appreciate this properly, we could more readily understand the fact that by this simple combination ninety-nine one-hundredths of all the cases in regard to the decay of teeth, etc., could be explained, each one individually. I do not think we will progress at all by debating in general about "activity of tissues;" "her health declined," etc. Beyond the principle of the survival of the fittest, I do not think there are any generalizations on that subject.

The experiments that have been begun in the line of organic alkalies are, in my opinion, investigations which lead up a little to that thing which humanity is striving after,—the elixir of life. Many of these organic alkalies are deadly and destructive. Take, for instance, the poison produced by hydrophobia,—one-twentieth of a grain produced the disease in a rabbit, and one-sixth of a grain of the poison taken from the brain killed a rabbit. If we can find such an active agent in the line of destruction, it is reasonable to suppose we can find just as active a power in the line of building up. We have only just begun our discoveries in the line of destruction. The investigation of this subject in the line of building up our bodies is not yet begun; the microbes of building up have not yet been investigated, but only those of destruction. Our whole bodies are essentially a collection of microbes. Every part seems to have a different set, and I believe that we shall yet find means and ways of cultivating the different microbes. It is within the line of possibilities that presently the cultivation of the microbes of the different organs will be a recognized industry, and by proper inoculation we shall be able to secure those for new kidneys, new brains, or anything of the kind. This is not a matter which has been created within the brain of the speaker by the extreme heat, for just as there are microbes of destruction, why should there not be those of construction?

*Dr. Charles A. Meeker.*—I would like to ask a very simple question, and sometimes it is the simple matters from which dentists derive a great deal of information.

Oftentimes our patients call upon us and say that their physicians have given them a preparation of iron,—an acid preparation, you might say; and we all know that in time this has a very disastrous effect upon the teeth. What preparation would you give to counteract the effect of that acid—what alkali? Would a solution of bicarbonate of soda be a good preparation to use in that case?

*Professor Mayr.*—That is certainly a very good one.

*Dr. Meeker.*—Do you know of a better one?

*Professor Mayr.*—In my opinion a physician has no right to so prescribe.

*Dr. Meeker.*—But sometimes they do.

*Professor Mayr.*—I would blame the physician then. There exist hundreds of preparations of iron which are alkaline, and in my opinion a physician exposes himself severely who prescribes an acid solution of iron. When there is two or three per cent. of muriatic acid in the stomach there is no need of an acid solution of iron. I think a physician ought to be given the thorough benefit of every possible blame for such a course. There are cases where pills made by Bland have been administered as a tonic. They consist of green vitriol, slacked lime, and a little bicarbonate of soda. They are entirely tasteless, and are easily dissolved in the stomach. It is a very effective preparation of iron, and I have used it in my own practice. In one case I gave a servant girl these pills; I made them large, thinking a servant girl would not appreciate anything which was very small. I put a little sugar in the mixture, and it looked real nice, and I told her to take three pills a day, and she took three pills three times a day without material injury. The iron is dissolved in the easiest manner, and I never saw a better preparation than that. I would certainly severely blame a physician who would prescribe an acid preparation of iron. That might have been done in the olden times, when they didn't know of any better means to be used against chlorosis than to dissolve iron nails in vinegar and drink it, but now we have such a number of alkaline preparations that I think those of an acid character have no place.

*Dr. Meeker.*—What was the iron given for?

*Professor Mayr.*—To color the blood and prevent chlorosis. As a fact, however, the blood in people suffering from chlorosis contains just as much iron as the blood in people having the dark-red fluid. The iron is there, but for some reason or other their red corpuscle factory, or spleen, makes only colorless blood-corpuscles, and it seems necessary to throw upon the organs that produce such corpuscles an extra amount of work, and then they make the red blood-corpuscles. The iron passes off; the amount absorbed is ridiculously out of proportion to the amount given, but while it passes through the system it is absorbed, has to be worked up, and that party of microbes whose business it is to make the red blood-corpuscles get all at once smothered with iron, and work to get rid of it, and thus get their activities aroused and finally get up to working-pitch. That is the office of the iron,—it merely arouses the red blood-corpuscles, producing activity, which an easy, normal course of life would not do. So also with morphia. If a person takes a quarter of a grain, it is absorbed in the system, and yet it can be recovered almost entirely in the urine. That shows what an amount of work may be done on a set of cells by the mere passage through them of a certain chemical. The mere passage of the morphia through certain cells in the brain produces a terrible amount of activity, although the morphia passes off. So it is with quinine. Ninety per cent. of it passes off without being damaged, and might be used over again; at the same time it has done its business.

*Dr. Peirce.*—It has been a whip?

*Professor Mayr.*—Yes, precisely.

*Dr. Meeker.*—Will the professor be kind enough to give us the prescription for that pill?

*Professor Mayr.*—I would use a drachm of sulphate of iron,—ordinary green sulphate of iron,—and one drachm each of carbonate of potash and starch, to be made into pills about, perhaps, one-sixth to one-quarter of an inch in diameter; the dose to be about three pills a day. That is entirely an alkaline preparation. The pills are spongy; they are not very solid.

*Dr. Meeker.*—How many pills would that make?

*Professor Mayr.*—Well, perhaps not a very great number,—just about enough for one case,—perhaps twenty or twenty-four pills. I generally use about that quantity. I use the metric system myself, and have to give it to you approximately in drachms. The preparation is very soluble, and has not the disagreeable effects on the intestines that follows the use of many fluid iron preparations. I have never known it to cause diarrhœa or anything of that kind.

*Professor Peirce.*—I have been very much interested in Professor Mayr's remarks. In the early part of his address he stated that there were certain acids which produced what we call erosion, some which left the surface rough and others which left it smooth. Where these acids leave the surface smooth, does the acid act also on the organic tissues as well as on the inorganic, and is that the reason the surface is left smooth?

*Professor Mayr.*—Certainly not. I think in that case a far more complex question than the mere action of acid arises. The question of polishing is a very important one. I do not think that acetic acid or lactic acid have a very powerful dissolving action on organic matters. Besides, the tendency of the tissue is to alkalinity and not to acidity. Yesterday I heard a statement made here that the natural condition of the tissues is that of acidity, but I myself should consider it one of alkalinity.

*Dr. Meeker*—What acid affects most rapidly the oxyphosphate fillings in the mouth?

*Professor Mayr.*—They are most usually affected by the crystalline acids; lactic acid affects them very powerfully, acetic acid not so powerfully. By changes of modes of living the different effects are shown. I had such a filling for fifteen years which kept perfectly well; it was on an open, exposed surface; it has never needed renewing. I had oxyphosphates in the front teeth that had to be renewed once every year. I think that these oxyphosphates suffered from something which has been mentioned during this meeting,—I mean abrasion. It came in contact with some gland which secretes either lactic acid or another crystalline acid, and dissolves rapidly. But the filling, which has kept perfectly well, also came in contact with a great deal of acetic acid in sour food, but did not dissolve.

*Dr. Meeker.*—We find the disintegration of the oxyphosphate in certain cases where it is in contact with the margin of the gum or quite near it?

*Professor Mayr.*—There the action would probably be that of lactic acid, because the lactic acid is found to be the acid in decay, and I presume that the lactic acid is frequently the acid which is at work in causing erosion and caries.

*Dr. Meeker.*—And the condition of the system would have some effect, inasmuch as it would produce more lactic acid in one than in another.

*Professor Mayr.*—I am very greatly in the dark there; I have found that it depends a good deal upon the surroundings. Not long ago I had to make a large amount of butyric acid, which is made by taking milk and chalk and yeast. They ferment together for about one year, and a very odorous fermentation is the result. During that time I noticed that all my gums became sore, my teeth were tender, and the membranes swelled up, presenting a somewhat scrofulous appearance. After I was through with the experiments these symptoms disappeared. I repeated the experiments and found I had only to go into that room where I made butyric acid to be infected with the germ. The germ of butyric acid seems to be the same as that of lactic acid, only another stage, and I think that the spores thrown out implanted themselves to an undue extent in my mouth, and my gums became sore. I found out in other ways that I had been actively infested by the germ of the acid. The air about me became strongly odorous; I presented the odor of old socks all the time. I could not be blamed for it; it was in my opinion a pronounced infection of lactic acid. There was a boy who worked in my office, and I had him in that room, and I said one day, "What is the matter with you? don't turn your mouth this way;" and he said "I cannot help it; I think I make the same odor that is in that pot." So he insensibly admitted to me that he was also infected with it. I think in many cases there may be infection with the germ of the lactic acid. After I stopped the experiment the whole train of symptoms totally disappeared, so much so that the doctor who had charge of my teeth noticed it. I think that very frequently such outside matters are the cause in those cases.

*Dr. Meeker.*—With the knowledge that the chemists have of the secretions of the oral cavity, can they improve on the oxyphosphate as a filling?

*Professor Mayr.*—The oxyphosphate is a filling that for practical purposes is very fine; but chemists who have time and leisure, and means and wits and brains and inventive skill, have been working for a long while on the idea of a filling that would resemble the teeth more closely and be stronger and harder than the oxyphosphate and not so easily affected as it is. But at the present time I think that the oxychloride is sometimes better than oxyphosphate. I have tried both and prefer the oxychloride. But you cannot give a general rule; in some mouths one would stand better than the other.

*Dr. R. McLean Sanger.*—Pardon me for digressing a little from what the professor has been saying; but last year, from the reading of his able paper the impression went forth that he would not recommend the application of the remedies of hypophosphite for the building up of the tissue, which is perfectly correct; but it is well established in medicine that many remedies, like iron, quinine, and mercury, are eliminated, or forced out of the system, just exactly as they went in. And so the thought came to me, in connection with his talk of last year, that possibly the benefits of the hypophosphite were not as tissue-builders, but as the "whip"

which he has already spoken of, to make the lower tissue-builders do their work. I speak of this to-day because for a year it has been bothering me, and perhaps some others of us labored under the same delusion,—that Professor Mayr meant to discourage the use of the chemical properties of hypophosphite as remedies; so I would like to ask him to-day to correct to the meeting, as he has to me already personally, that impression.

*Professor Mayr.*—In regard to this hypophosphite, I will say that I read the report of my address, and noticed also that it did give somewhat the impression that I did not consider them of value. I do consider them of great value as a stimulant, as a tonic; but of no value whatever as tissue-builders. That is to say, the atom of hypophosphites introduced into the system, four hours afterwards will be found as such in the urine; but while it has gone through the body it has done a vast amount of stimulating and arousing; it has whipped somebody—it did some business. I noticed that a theory was propounded that the hypophosphites had changed to phosphates. To a chemist that is a most untenable theory. The body itself is not only a very poor oxydizer, but it is ever a powerful reducer. I should rather expect indeed to see the reverse effect; as a rule there will be no oxydization to speak of, and hypophosphites do not oxydize so very easily.

*Dr. Meeker.*—Does this apply to Horsford's preparations?

*Professor Mayr.*—The Horsford preparations I do not consider very good, for the reason that they are but little stimulant to the body and are certainly not assimilated. I have made experiments and found that every grain of acid phosphate taken into the system is thrown out with very great rapidity. It is not needed in the system. So these acid phosphates may be a palatable drink, just like a great many other things that we take, but I do not think they are, as a rule, of the least value as a tonic or otherwise; quite to the contrary, they demoralize. The stomach does not want too much mineral acid food. If you want to get a good acidity of the stomach, put bicarbonate of soda in it, and it will pour forth volumes of hydrochloric acid, while, on the other hand, if you put a mineral acid into the stomach, it diminishes its acid-producing qualities; this is simply on the principle of general natural obstinacy. It don't want to do the thing which you think natural for it to do; but try to make it go the other way, and it will do just that very thing.

*Dr. Sanger.*—What preparation would you suggest as a good one where hypophosphates are deemed to be necessary?

*Professor Mayr.*—The phosphates that are needed in the system can only be taken in their natural combination with albumen, as in grain food. I do not think we have any preparation of a phosphate that is assimilated, excepting that phosphate which is combined with albumen in grain and meat. The gristle in pigs' feet, for instance, contains a great deal of very assimilative phosphates; they are ready to be assimilated, they have not become bone yet. I do not think we have any chemical preparation whatever that is as assimilative in the line of phosphates.

*Dr. Meeker.*—I should like to ask the professor about something which has always been curious to me, and that is, what acid there is in asparagus and what effect it has on the system as it goes through the kidneys?

*Professor Mayr.*—Well, there is a compound in asparagus called asparagine—asparaganic acid and asparagine; the peculiar change it undergoes is as unknown as that of turpentine when inhaled. You can in the laboratory change the odor of turpentine into an oil having an odor somewhat of violets, but it is very uncertain whether anything like it is going on in the kidneys.

*Dr. Meeker.*—Do you know whether it has any effect upon the blood? People seem to think it has.

*Professor Mayr.*—Yes, they have an idea that the odor which follows its use was somewhere hidden up in the system, and for that purpose in France a great remedy for all kinds of diseases is a kind of black radish which they cultivate there to a very great extent, and which creates a somewhat pungent odor, and they think that when the smell comes off with the eructations it is taken somewhere out of the system. I think when the smell comes off the odorous matter is in the asparagus or the radish, and that by meeting with some other chemical in its passage through the system the peculiar odorous matter is somehow or other made out of the asparagine.

*Dr. J. A. Osmun.*—I would like to ask you, as you were speaking of acetic acid being an active principle of decomposition, and also as producing caries, whether it is possible to produce a mouth-wash which will kill the microbes or retard the action of caries?

*Professor Mayr.*—I think that there you will meet with the same perversity of nature that you have in the stomach, and the more alkali you put on the glands the more they will pour out; by the principle of natural perversity you will find that no success will be reached, for by trying to neutralize it by an alkali you will find they will all be the more active and work the harder. I think that a good preparation is something containing a little salicylic acid. Most of you have heard of the very disagreeable odor arising from sweaty feet. In that case there is an excess of butyric acid; that is what causes the odor. Soda won't help it very much, but very mild sulphuric acid is a very good remedy indeed. An alkali does not remove it at all. The other day a gentleman brought me a little box of a French preparation for which he paid five dollars. I examined it, expecting to find some powerful chemicals in it. It was powdered alum, worth two cents a pound; and it worked excellently. It is an acid preparation. In that case an acid preparation prevented those glands from giving acid secretion. I should infer that salicylic acid, if properly used, would be a good remedy. That is not an acid really; it has no solvent powers whatever.

*Dr. Meyer*, of Baltimore.—I am only here for pleasure, and not so much to take part in this meeting; but I should like to ask the professor to say something about cocaine and its action upon the nerves.

*Professor Mayr.*—In that case I have to confess my ignorance. Of course we all know the outward effects, but the details are entirely unknown in regard to cocaine. We all know its superficial anæsthetizing effects, but it is entirely unknown by what process or for what reason the effects are produced.

*Dr. Newton.*—The professor says that the carmine test is very successful; I would like to know how to use it.

*Professor Mayr.*—I take the cochineal bugs and grind them up in a mortar, with a little alcohol and water, and the solution thus produced keeps perfectly well. Put a little fresh on neutral paper and use it immediately. Litmus paper is affected by carbonic acid, but carmine is not. If I have a solution of bicarbonate of soda and I put a few drops of acid in that, your litmus paper will show apparent acidity; it is no longer reliable; but, supposing I use carmine, it will remain blue until the last drop of bicarbonate of soda has been neutralized. I have made probably five thousand tests, and I find that the reaction of the urine is no value whatever. Within two hours the acidity will be changed to alkalinity, and, by the presence of a little sugar, to that of acidity. I find a great many physicians put stress on the reaction of urine, but it is not of any value at all in my opinion.

